# Update on Diagnosis and Treatment of Uveitic Glaucoma

**DOI:** 10.3390/jcm13051185

**Published:** 2024-02-20

**Authors:** Ioannis Halkiadakis, Kalliroi Konstantopoulou, Vasilios Tzimis, Nikolaos Papadopoulos, Klio Chatzistefanou, Nikolaos N. Markomichelakis

**Affiliations:** 1 Ophthalmiatrion Athinon, Athens Eye Hospital, 10672 Athens, Greece; kalikonsta@yahoo.gr (K.K.); tzimisv@hotmail.com (V.T.); papadopoulosn1992@gmail.com (N.P.); 2 First Department of Ophthalmology, National and Kapodistrian University of Athens School of Medicine, Athens General Hospital “G. Gennimatas”, 11527 Athenbs, Greece; kliochat@med.uoa.gr; 3 Ocular Inflammation Institute of Athens, Sarantaporou 7 Agios Stefanos, 14565 Athens, Greece; nmarkom@otenet.gr

**Keywords:** uveitic glaucoma, inflammatory glaucoma, CMV, minimally invasive glaucoma surgrery, Ahmed valve, Baerveldt tube

## Abstract

Glaucoma is a common and potentially blinding complication of uveitis. Many mechanisms are involved alone or in combination in the pathogenesis of uveitic glaucoma (UG). In terms of diagnostic evaluation, the effects of inflammatory activity in the retinal nerve fiber layer may be a source of bias in the interpretation of optical coherence tomography measurements. For the successful treatment of UG, the control of intraocular inflammation specific to the cause or anti-inflammatory treatment, combined with IOP management, is mandatory. The early institution of specific treatment improves the prognosis of UG associated with CMV. The young age of UG patients along with increased failure rates of glaucoma surgery in this group of patients warrants a stepwise approach. Conservative and conjunctival sparing surgical approaches should be adopted. Minimally invasive surgical approaches were proved to be effective and are increasingly being used in the management of UG along with the traditionally used techniques of trabeculectomy or tubes. This review aims to summarize the progress that recently occurred in the diagnosis and treatment of UG.

## 1. Introduction

Uveitis is the most common inflammatory eye disease, with an incidence of 17–52.4 cases per 100,000 population [1,2,3,4,5,6,7]. The incidence of secondary glaucoma caused by uveitis is reported to be 10–20% [8,9,10,11]. Daniel et al. reported that in adults with non-infectious uveitis, mean annual incidence rates for ocular hypertension (OHT) with intraocular pressure (IOP) ≥ 21 mmHg and IOP ≥ 30 mmHg were 14.4% and 5.1% per year, respectively [12]. Furthermore, OHT in uveitis, in contrast to POAG, progresses rapidly to uveitic glaucoma (UG) [13]. Uveitic glaucoma is aggressive, with a high likelihood of requiring surgical management and a high risk of central vision loss. Glaucoma is the most common cause of permanent vision loss in cases of anterior uveitis, accounting for 30.1% of cases with moderate visual loss [14,15]. Certain types of uveitis carry a higher risk of developing secondary glaucoma; namely, Posner–Schlossman syndrome (PSS), herpetic uveitis, Fuchs heterochromic iridocyclitis and juvenile idiopathic arthritis. Risk factors which increase the incidence of UG apart from etiology are race, age, duration of inflammation and steroid use [6,9,16,17].

In cases of UG, a successful clinical course implies prompt and effective treatment of uveitis, high suspicion and early identification of glaucomatous changes and aggressive control of the IOP. Management can be particularly challenging with medical therapy alone; meanwhile, the success of glaucoma surgery is lower in these patients compared to the general population, with a higher incidence of postoperative complications [13,18].

## 2. Pathophysiology of Uveitic Glaucoma

Several mechanisms may be responsible (alone or in combination) for the occurrence of glaucoma in uveitis patients.

**Figure 1:**
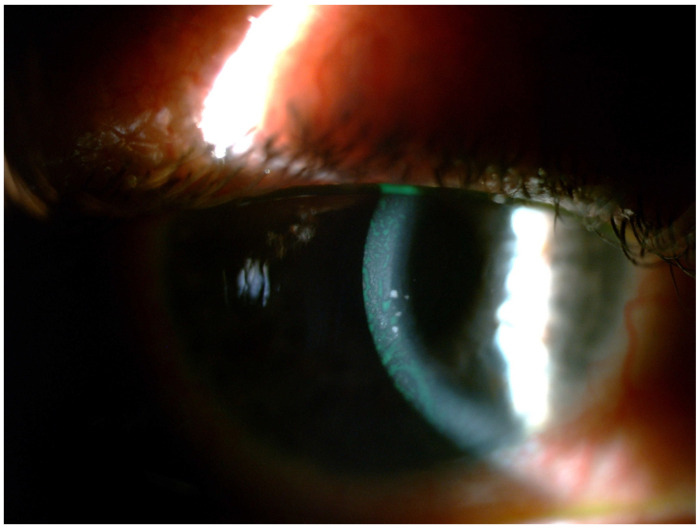
CMV anterior uveitis with characteristic large keratic precipates.

**Figure 2:**
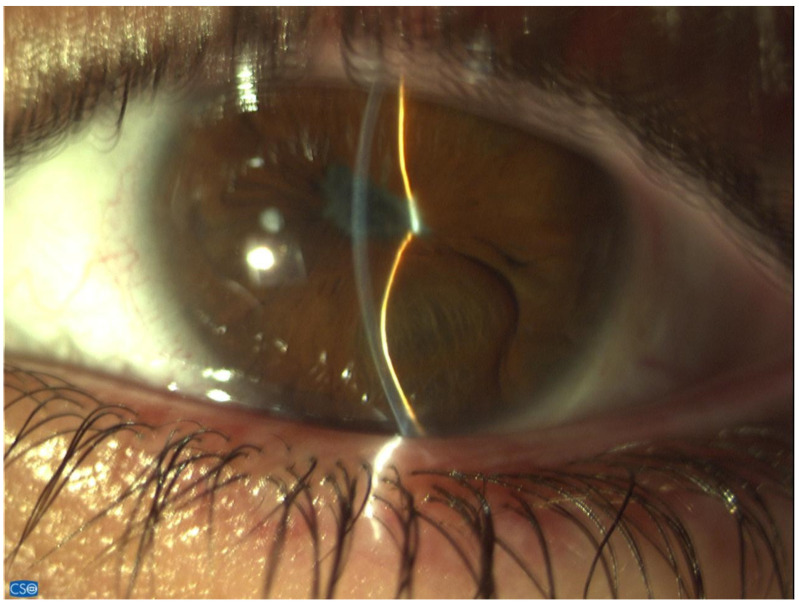
Angle-closure glaucoma with iris bombe.

1. Alteration of aqueous humor consistency and reduced permeability of trabecular meshwork: The breakdown of the blood aqueous barrier during inflammation results in higher concentrations of proteins in aqueous humor. This transudate may not in all cases of uveitis immediately affect pressure; however, over time its accumulation at the trabecular meshwork reduces drainage rate [6,19]. Trabecular precipitates in the form of circulating inflammatory cells and debris may further clog the Schlemm’s canal and reduce drainage.
2. Inflammation of the trabecular meshwork (trabeculitis) resulting in thick and edematous trabecular filaments, as well as the accumulation of fibrin and inflammatory cells in the outflow channels (as shown in herpetic and cytomegalovirus trabeculitis) produces significant obstruction to drainage and extremely high IOP Figure 1 [20,21]. Granulomas in the angle in cases of granulomatous uveitis may also impair drainage.
3. Acute angle-closure pupillary block: The formation of synechiae posteriorly between the iris and the lens may lead to seclusion of the pupil, forward iris bowing with apposition to the angle and angle-closure glaucoma (Figure 2).
4. Secondary acute angle closure: swelling and anterior rotation of the ciliary body, as well as choroidal effusion, can lead to angle obstruction.
5. Chronic angle closure: Anterior synechiae formation between the iris and the angle due to the increased coagulative state of the inflamed iris may cause chronic angle closure [22]. Recently Alvarez Guzman et al. reported that the majority (80%) of cases of glaucoma associated with Vogt–Koyanagi–Harada disease were due to angle closure [23]. In the event of a uveitis that causes significant retinal or ocular ischemia, pronounced neovascularization can affect the trabeculum, with inevitable aqueous flow obstruction and intractable glaucoma [24].
6. Special consideration should be given to the impact of corticosteroid use in glaucoma, as their use in effectively controlling the inflammation is a double-edged sword. Steroids may be the cause of UG in up to 42% of cases [10,25]. Common risk factors for steroid response in uveitis are primary open-angle glaucoma, familial history of glaucoma, rheumatoid arthritis, extremes of age (children and the elderly) and diabetes [21,26,27]. It becomes evident that in uveitis, glaucoma may present either with an open angle or with an angle-closure mechanism, or even with a combination of both.

There are also poorly studied mechanisms involved. For example, an attempt to identify a causal association between uveitis and glaucoma in the general population has disclosed a possible genetic link [28].

## 3. Diagnosis

High vigilance for early signs of IOP elevation and optic nerve damage in patients with uveitis is important.

**Figure 3:**
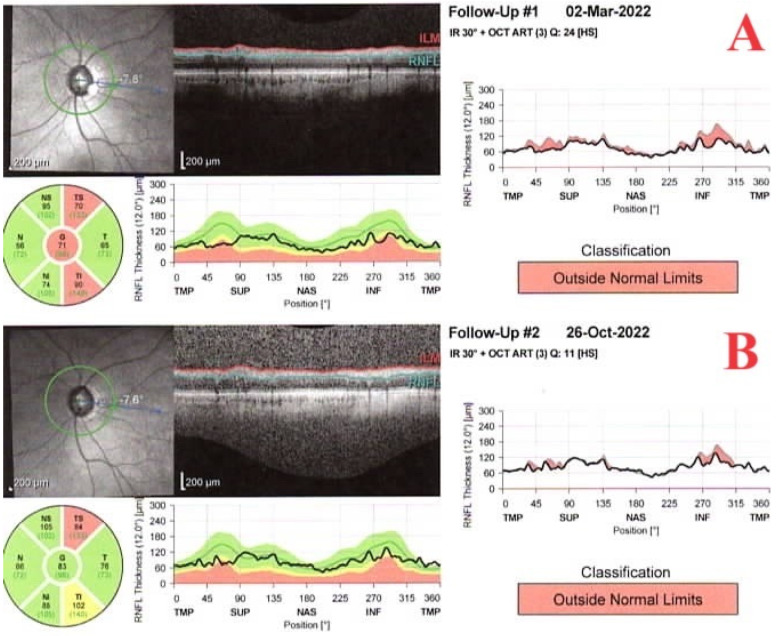
Retinal nerve fiber layer thickness before (**A**) and during (**B**) inflammation.

Sequential visual field tests and optical coherence tomography (OCT) tests with attention to the retinal nerve fiber and ganglion cell layer thickness are mandatory. However, diagnosing glaucomatous changes during episodes of inflammation can pose a significant challenge to the ophthalmologist, as active uveitis may influence the results. Studies have revealed that in patients with active uveitis and no glaucoma, OCT displays a thickened retinal fiber layer (RNFL) compared to healthy individuals. The RNFL thickness may remain normal even in eyes with quiescent uveitis and early glaucoma. Therefore, RNFL thickness measurements should be interpreted cautiously, and screening for glaucoma is best performed when the eyes are going through periods of quiescence [29], as shown in Figure 3. The measurement of blood flow by OCT angiography is an additional method for the detection of early glaucomatous lesions. The vessel density in the area of the optic nerve head and the macula has been shown to be reduced in primary open angle glaucoma (POAG). Several studies have indicated that the peripapillary vessel density and RNFL thickness have a similar sensitivity and specificity in diagnosing POAG [30]. In the sole study to date evaluating the use of OCT angiography in UG, Liepsech et al., reported that vessel density was reduced in the area of the optic nerve head and the macula in UG eyes in comparison to normal eyes [31]. Another case report verified the use of OCT angiography in diagnosing glaucoma in an instance of active uveitis [32].

Additionally, anterior-segment OCT (AS-OCT) may assist in revealing corneal thickness, abnormal irises, the ciliary body and angle configuration; this will lead to early intervention. At the same time, if the cornea precludes visualization of the anterior segment, ultrasound biomicroscopy can offer valuable information regarding the anterior segment structures. Importantly, these ancillary tests should never negate or replace regular and meticulous clinical examination and gonioscopy; this is crucial for these patients, for whom persistent inflammation, increasing pigment deposition, granulomas, posterior or anterior synechiae and early neovascularization of the angle may be identified and treated accordingly. Gonioscopy should always be performed to identify the presence of synechiae nodules or neovascularization in the angle and to establish the extent of angle closure [22].

## 4. Treatment

Management of UG is multidisciplinary and involves both strict control of the inflammation and treatment of the IOP elevation. Treatment can be both medical and surgical.

### 4.1. Medical Treatment

Identifying the cause of uveitis is crucial, as in cases of infectious etiology, where treating the underlying infection will expedite resolution of the disease. This is best exemplified in herpetic or toxoplasmic uveitis, where antiviral and antiparasitic medication produce an immediate improvement to the clinical signs and symptoms [33,34]. It has been shown that IOP in CMV-positive PSS is more difficult to control than in CMV-negative cases. Treatment of CMV infection with valganciclovir or ganciclovir has been shown to improve PSS control [35] and contribute to withdrawal of the steroids in cases of steroid-dependent PSS [36]. Touhami et al. have found that in cases of CMV anterior uveitis, the institution of early (<700 days) antiviral treatment reduced the need for later antiglaucoma surgery [37]. This is attributable to the fact that early antiviral treatment prevented permanent damage to the trabecular meshwork.

In the event of idiopathic or immune-mediated diseases, corticosteroids (administered either locally or systematically), immunosupressants or the recently introduced monoclonal antibodies should be used accordingly. Treatment should be aggressive, aiming to achieve quiescence; furthermore, one should not choose to undertreat in order to avoid corticosteroid-induced IOP rise, as the adverse effects of chronic inflammation may further complicate the outcome [6,27]. However, great consideration should be given to the mode of implementation of steroid treatment. A recent multicenter study has shown that eyes treated with fluocinolone implant have substantially higher risk of developing glaucoma than eyes treated with systemic therapy (40% vs. 8% in 6.9 years) [38].

B-blockers, prostaglandin (PG) analogs, a-adrenergic agonists, topical and systemic carbonic anhydrase inhibitors and combined preparations may be used for the control of IOP in cases of UG. There is controversy regarding the use of prostaglandin analogues (PGAs) as first-line agents in UG due to their proinflammatory properties, the possibility of exacerbation of herpes simplex keratouveitis and the occurrence of cystoid macular oedema [39]. There are studies that support their safety for UG patients [40], and some authorities advocate the use of PGAs as first-line treatment [41,42]. Furthermore, there are indications that bimatoprost has a much lower propensity in causing uveitis or macular edema than latanoprost [43]. On the contrary, cholinergic agonists are usually avoided in uveitic patients, as they are proven to aggravate inflammation (by increasing blood aqueous barrier breakdown) and promote posterior synechiae formation.

Investigations for effective treatment are ongoing and ripasudil, a rho-kinase inhibitor that was first introduced in Japan in 2014 has demonstrated effectiveness in approximately 50% of patients suffering from glaucoma [44,45]. Recent studies suggest that ripasurdil is particularly effective in eyes with ocular inflammation that receive steroids, as it may have an anti-inflammatory effect along with its effects to the IOP [45,46].

### 4.2. Laser Treatment

Nd-YAG laser peripheral iridotomy (LPI) is used for anterior chamber angle closure due to posterior synechiae and iris bombe, but it is not always successful in UG. According to the sole study to date that evaluated the results of LPI in acute angle closure secondary to UG, 62% of LPIs performed did not remain functional after 85 days. Therefore, the performance of at least two iridotomies and intensive treatment with corticosteroids and cycloplegics is recommend. The performance of LPI should be avoided in eyes with severe active anterior uveitis, corneal oedema or iridocorneal touch, as a shallow anterior chamber increases the risk of endothelial damage during LPI [22,47].

Argon laser iridoplasty was successful in one case of acute angle closure associated with uveitis that did not respond to repeated LPIs and medical treatment [48].

Until recently, SLT was not considered a treatment option for UG because of the inflammation that it may induce [49]. However, recent publications tend to refute this theory. Initially Maleki et al. performed SLT in 15 eyes of 14 patients with stable uveitis who had received one fluocinolone implant that caused glaucoma. Their success rate at 1 year was 46.7%, slightly less favorable than in patients with POAG [50]. Xiao et al. performed high-energy (1.2–1.5 mJ per pulse as opposed to 0.9 mJ for regular treatment) SLT treatment in 20 patients with steroid-induced glaucoma and quiescent uveitis and reported a 65% success rate without complications. A more frequent postoperative steroid regimen was followed [51]. Recently, Zhou et al. compared the reduction of IOP and complications after SLT in UG and POAG or PEX glaucoma. They did not find a difference except at a time point 3–8 weeks after treatment [52]. At this time point, the reduction of IOP was greater in the UG group than in the PEX glaucoma group. In conclusion, even though data are limited, it seems that SLT is a promising treatment which can be applied in quiescent uveitis cases with steroid-induced glaucoma.

Cyclodestructive procedures using the 810 nm diode laser are most of the time reserved for cases in which all other methods of surgical treatment have failed. Because of the very serious complications they cause, they are a final choice in UG. Applying transcleral diode laser cyclophotocoagulation (TD-CPC) to an already inflamed and underactive ciliary body can cause severe damage. Laser cyclophotocoagulation may cause severe hypotony in 19% of patients and is likely to cause phthisis, irreversible anatomical lesions to the globe and loss of vision [53]. However, in a small series of 20 patients using a treatment mode of 10–15 applications of 2.0 W energy applied for 2 s to treat no more than 270°, Shlote et al. reported a 72.2% success rate without any serious adverse effect [54]. Voykov et al. used TD-CPC to treat 16 patients with Fuchs uveitis. In 10 of them, TD-CPC was the sole surgical treatment. After 1 year, control of IOP was achieved in 6 out of 10 patients (60%). There was no exacerbation of intraocular inflammation, no postoperative hypotony and no phthisis bulbi in the 16 patients who underwent CPC [55]. In contrast, Heinz et al. used TD-CPC to treat UG attributed to juvenile rheumatoid arthritis and reported a 32% qualified success after 9 months [56].

Recently, micropulse wave transscleral diode cyclophotocoagulation has been proposed as an alternative to TD-CPC, offering a better safety profile. Its operating principle is based on short laser pulses (ON cycles) separated by intervals corresponding to the thermal relaxation time (OFF cycles). During ON cycles, energy accumulates in the pigmented epithelium to achieve the coagulation threshold. It has been proposed that the OFF cycles allow thermal dissipation and thus reduce collateral damage and adverse effects such as inflammation and chronic hypotony. Several studies reporting favorable results with micro-pulse diode cyclophotocoagulation included a small number of eyes with UG [57,58].

### 4.3. Surgical Treatment

According to several studies, almost 30% of patients with UG will need surgical treatment [8,59,60]. This percentage may be significantly higher in children [8]. Surgical treatment of UG may be challenging for a variety of reasons. Persistent intraocular inflammation, extensive use of steroids and extreme IOP range are factors that need to be considered when choosing the appropriate surgical technique. Uveitis patients may have a wide variation in their IOP and there is always a possibility of ocular hypertension alternating with ocular hypotony, with devastating consequences for the eye. While reviewing the literature of more than two decades, it is generally accepted that either trabeculectomy [59] or valve [60] implantation are safe and most of the times successful procedures in the treatment of UG. However, most of studies evaluating surgical techniques in UG are retrospective in their design with a small number of participants; furthermore, they present data in various different ways, and many of them have a limited follow-up period. Given the limited lifespan thatraditional glaucoma surgery has and the fact that UG patients who present for glaucoma surgery are either in their mid-fifties or children, alternative conjuctival sparing approaches are being considered. Recently, various techniques of minimally invasive glaucoma surgery (MIGS) are being instituted in the surgical treatment of UG. Most of the instances are performed as primary procedures or along with cataract surgery, but some are tried after other methods have failed.

## 5. Trabeculectomy

Trabeculectomy has been the preferred surgical procedure for UG for many years [61,62]. Although studies evaluating the success rate of trabeculectomy in UG are retrospective with a small number of patients, most of them agree that the success rate of trabeculectomy with MMC is reduced in UG in comparison to POAG [63]. There are at least two reasons for this: Inflammatory activity is likely to be more pronounced in uveitic eyes following intraocular surgery, leading either to hypotony due to ciliary body impairment or to bleb failure due to subconjunctival scarring. Almarobac et al. reported that the cumulative probabilities of success were 60% and 35.7% at 36 and 60 months postoperation, respectively, whereas in the largest up-to-date series, Iwao et al. reported probabilities of success 1, 3 and 5 years after trabeculectomy in the UG group of 89.5%, 71.3% and 61.7%, respectively [63,64]. In a recent study, Kanaya et al. reported that the success rates in UG and POAG were 91.7% and 88.0% at 12 months, 82.2% and 75.6% at 36 months, and 66.5% and 61.8% at 120 months, respectively [65]. The authors attributed the increased success rate of trabeculectomy in UG (which was similar to that in POAG) to the successful control of the inflammation. Different studies evaluated risk factors for the failure of trabeculectomy in cases with UG. Iwao et al. considered cataract surgery and granulomatous uveitis as a risk factor for failure [63]. Almobarac et al. reported that in UG eyes that underwent phacoemulsification following MMC-enhanced trabeculectomy, the bleb survived but the eyes required more medication to control the IOP after the procedure [66]. In contrast to Iwao et al. [63], Kanaya et al. reported that granulomatous uveitis was significantly associated with favorable prognosis [65]. There is controversy regarding whether preoperative inflammation affects the results of surgery. On the contrary, most studies agree that postoperative inflammation is a risk factor of worsening failure rate for trabeculectomy surgery [67,68].

Kwon et al. looked specifically at the effect of the activity of inflammation on the success rate of trabeculectomies in UG and concluded that the initial activity of inflammation did not affect the success rate, but relapses of the inflammation were risk factors for failure [69]. In contrast, recently, Magliya et al. concluded that proper perioperative uveitis control in patients attending UG surgeries results in lower IOP levels and less inflammation over 2 years postoperatively [70]. Finally, Gregory et al. evaluated the effect of race on the course of UG and concluded that trabeculectomy has a higher risk of failure in black patients [71].

## 6. Minimally Invasive Glaucoma Surgical (MIGS) Devices in Uveitic Glaucoma

Minimally invasive glaucoma surgical (MIGS) devices have been developed as a surgical option for glaucoma, to improve surgical safety, conserve conjunctiva and maintain efficacy in terms of lowering IOP. Procedures that disrupt, ablate, or bypass the trabecular meshwork (TM) constitute MIGS. These procedures include ab interno trabeculectomy using the Trabectome^®^ (NeoMedix, Tustin, CA, USA), goniotomy with the Kahook Dual Blade^®^ (New World Medical, Cucamonga, CA, USA) and gonioscopy-assisted transluminal trabeculotomy (GATT). These techniques are blebless and target the TM, the primary anatomic structure responsible for aqueous outflow resistance.

Goniotomy has traditionally been used to treat pediatric UG. It has been shown that it results in a significant decrease in IOP and number of IOP-lowering medications, although multiple interventions are often needed [72,73]. Recently, Meerwijk et al. reported success rates of 100%, 93% and 80% at 1, 2 and 5 years, respectively, after performing goniotomy in children with a mean age of 7 years and non-infectious UG [74]. There were no significant changes in visual acuity and uveitis activity or its treatment, and there were no major complications.

Trabectome (Neomedix, Tustin, CA, USA) is a MIGS device that uses electrocautery, irrigation and aspiration to selectively ablate the trabecular meshwork and the inner wall of Schlemm’s canal and allow aqueous free access to the canal and its collector channels. Anton et al. used Trabectome to treat 24 patients with UG and reported that there was no patient who achieved absolute success but 87.5% of the patients achieved qualified success after 1 year. Three (12.5%) patients needed further glaucoma surgery [75]. According to Swamy et al., who reported the results of the operation from 45 eyes with UG from the Trabectome Study Group database, the qualified success rate at 12 months was 91%. Six (13.4%) cases required secondary glaucoma surgery and no other serious complication were noticed [76].

The Kahook dual blade (KDB) is a disposable handpiece that employs two parallel blades to remove a strip of trabecular meshwork to improve outflow. It may be combined with phacoemulsification. Murata et al. performed ab interno trabeculotomy with KDB in 24 eyes with UG and reported that the success rate was 33% in 1 year [77]. In contrast, Chen et al. performed KDB in 24 eyes of 22 patients and reported an 86% success rate after 2 years [78]. TrabEx+ (MST, Redmond, WA, USA) consists of a handpiece with a dual blade that is also connected with an irrigation and aspiration system that adapts to each machine for phacoemulsification. Tanev et Kirkova reported 100% qualified success 18 months after performing TrabEx in 12 patients with UG [79].

Gonioscopy-assisted transluminal trabeculotomy (GATT) is a minimally invasive ab interno procedure that has evolved from traditional trabeculotomy techniques and is performed with a prolene suture or with the guidance of an illuminating micro-catheter device. The surgical procedure involves cutting through the trabecular meshwork, cannulating the Schlemm’s canal 360° and unroofing the Schlemm’s canal. GATT is believed to reduce IOP by fracturing the trabecular meshwork and removing the resistance to aqueous outflow. Initially, Sachdev et al. successfully used GATT in three young patients with JRA uveitis [80]. Very recently, Gunay et al. reported favorable results in two other patients [81]. Parkish et al., in a small study of 16 eyes with uncontrolled UG, reported a cumulative success rate of 81% at 12 months. Transient hyphema was seen in 44% of eyes [82]. In the largest series to date, Belkin et al. used GATT in 33 eyes of 32 patients with UG who underwent GATT with or without concomitant cataract extraction. Surgical success was achieved in 71.8% of cases in 1 year. No sight-threatening complications occurred during surgery or follow-up [83]. Sotani et al. performed microhook trabeculotomy with a straight Tanito microhook (M-2215 s, Inami & Co., Ltd., Tokyo, Japan) in 36 eyes of 30 patients and reported that after 1 year, surgical success was achieved in 67% of eyes [84]. Other MIGS used are the i-stent and Hydrous, which might have a role as primary or secondary conjunctival sparing procedures in UG [85].

### Bleb-Forming Devices

Similar to traditional filtering surgery, another MIGS approach to reducing IOP is to shunt aqueous from the anterior chamber to the subconjunctival space. The Xen^®^ gel stent (Allergan INC, Dublin, Ireland) and PreserFlo^®^ (Santen, Osaka, Japan) microShunt utilize this approach. Because this approach results in the formation of a filtering bleb, there is debate as to whether they may truly be classified in the MIGS category.

The Xen implant stent is a hydrophilic tube that is 6 mm long with a lumen of 45 μm, and it is composed of porcine gelatine crosslinked with glutaraldehyde to prevent degradation when implanted [86]. In 2018, Sng et al. published the first results with Xen-45 in UG. They implanted Xen-45 in 24 consecutive UG patients, in the majority of whom conventional glaucoma surgery was considered inevitable. The 12-month cumulative Kaplan–Meier survival probability was 79.2% [87].

Qureshi et al. performed Xen-45 implantation in urgent basis in 37 eyes with uncontrolled glaucoma. At the end of the follow-up period (12 to 23 months; mean: 16.7 months) five eyes (13.5%) failed, needing further glaucoma surgery. The cumulative probability of absolute success was 89.2% 1 year after surgery [88].

Recently, Evers et al. reported results for Xen-45 implantation in 25 eyes with uncontrolled UG. Six eyes (24%) underwent surgical revision and were considered failures. At the final follow-up (mean: 17.7 months), 72% of eyes achieved complete success and 4% of eyes qualified success. Notably, the Xen implant did not prevent IOP spikes during uveitis activity [89].

Serar et al. reported the successful use of Xen-63 with a larger lumen in the case of a refractory neovascular glaucoma due to Fuchs heterochromic iridocyclitis and retinal vein occlusion after the failure of an Ahmed tube. After one-year, intraocular pressure was 16 mmHg without any medication and the bleb was well-formed [90].

The PreserFlo^®^ microShunt is an 8.5-mm-long glaucoma filtration surgical device with a 350 μm outer diameter and a 70 μm lumen that is implanted through an ab externo technique. The device’s proximal tip rests in the anterior chamber while the distal tip sits under the conjunctiva and Tenon’s capsule, about 6 mm beyond the limbus, enabling aqueous humor to pass through the lumen to produce a posterior bleb after implantation [91]. Triolo et al. reported 36-month results of PreserFlo implants in a consecutive series of 21 patients with UG. The mean rates of success were 68%, 47% and 47% at 12, 24 and 36 months postoperation, respectively [92].

## 7. No Penetrating Glaucoma Procedures in Uveitic Glaucoma

In uveitic patients, non-penetrating surgery offers the advantage of minimal post-operative anterior chamber inflammation and a reduced risk of delayed complications such as hypotony and bleb-related infections, which are more common with trabeculectomy. The absence of an iridectomy and anterior chamber penetration is supposed to reduce the inflammatory response while the presence of a trabecular meshwork may act as a barrier to infectious organisms entering the eye. Satisfactory long-term results have been reported for non-penetrating glaucoma procedures (deep sclerectomy (DS) and viscocanalostomy) in the management of UG. Obeidan et al. performed DS in 33 consecutive eyes of 21 patients and after a mean follow-up of 33.2 months reported that complete success was obtained in 72.7% of eyes, whereas qualified success was obtained in 21.2% of eyes, yielding an overall success rate of 93.9% [93].

Mercieca et al. reported that after performing DS with 0.2–04 mgr/l MMC in 43 eyes of 43 patients, the probabilities of IOP < 22 mmHg and <19 mmHg were 69% and 62% at 3 years and 60% and 51% at 5 years, respectively. Most eyes (60%) had a Nd:Yag laser goniopuncture (LGP) by the fifth year. Recurrence of uveitis was observed in 16 eyes. Seven eyes (16.3%) had subsequent glaucoma procedures [94].

The limitation of deep sclerectomy is that it is technically difficult to perform manually, which has limited its popularity. CO^2^ laser-assisted sclerectomy surgery (CLASS) is an improved version of DS that uses a CO^2^ laser, which is precise and easily strips the deep sclera, unroofs the Schlemm’s canal (SC) and leaves the trabecular meshwork thin enough for aqueous humor percolation. Xiao et al. performed CLASS in 22 eyes with UG and in 25 eyes with POAG and compared the results. After 1 year, the qualified surgical success was comparable between the UG (86.9%) and POAG (96.0%) groups, and the complete success rates were 60.9% and 64.0% in the UG group and POAG group, respectively [95].

Recently, Salloukh et al. presented long-term results after performing vicocanalostomy in 16 patients with UG. Complete and qualified success was seen in 75% and 94% of patients at year 1, 50% and 86% of patients at year 3 and 19% and 75% of patients at year 5 [96]. The long term (>2 years) outcomes of the aforementioned procedures, according to the recent literature, are presented in Table 1.

## 8. Tube Shunt Surgery in Uveitic Glaucoma

Tube shunt (aqueous shunt) surgery has traditionally been reserved for refractory glaucoma. Therefore, shunts are commonly performed as primary surgery in UG.

Ahmed Valve and UG: The Ahmed Glaucoma Valve^®^ (AGV, New World Medical Inc., Rancho Cucamonga, CA, USA) is a glaucoma drainage device commonly used for the treatment of glaucoma. It can be used as a primary surgical procedure or after failure of a previous filtration procedure. As the AGV has an internal valve mechanism consisting of thin silicone elastomer membranes, it does not require additional restrictive mechanisms to limit aqueous humour flow from the anterior chamber to the subconjunctival space. The above valve mechanism prevents early hypotony, which is considered advantageous, especially in UG. The body plate is usually placed 8–10 mm from the limbus while the tube is inserted 2–3 mm into the anterior chamber (AC), sulcus or even the vitreous cavity, depending on AGV type. More recently, a new type of AGV was introduced (Ahmed ClearPath GDD) which lacks the internal valve mechanism. It is available in 250 mm^2^ and 350 mm^2^ sizes and a pre-threaded 4–0 rip cord is provided by the manufacturer to prevent hypotony during the early postoperative period.

Data for more than 20 years can give us a general idea of what to expect when using these shunts as far as outcomes and complications are concerned. Earlier studies indicated that a significant IOP reduction (at least 25% from preoperative values) was achieved in more than 70% and 50% of patients at 1 and 4 years postoperation [97,98,99]. A significant reduction of glaucoma medications was also detected in all cases, but up to 17% of eyes experienced complications during the follow-up period [97]. The most important complications were tube occlusion, valve exposure and corneal decompensation [97,99]. It has been proposed that sulcus placement of the tube is associated with a moderate decrease in endothelial cell count and is strongly recommended in eyes at high risk of corneal failure. Macular edema as well as ocular hypotony is still a concern even with the use of AGV. Ramdas et al. reported that 13.2% and 15.8% of UG patients developed macular edema and hypotony, respectively, after AGV and Baerveldt-350 implantation. These percentages were higher compared to non-uveitic patients, but the difference was not statistically significant. IOP reduction was comparable to that of non-uveitic glaucoma patients (44.9% vs. 42.8% decrease) [18]. When AGV performance was evaluated as the mean IOP decrease postoperatively, this was ranged from 11 mmHg to 25.2 mmHg [100,101,102,103,104]. A mean decrease in the number of antiglaucoma medications was also achieved (1.88) [100,101,102,103,104]. Combining AGV with fluocinolone implant resulted in even less need for glaucoma medications [105]. The success rate was relatively higher in eyes with pars planitis and lower in eyes with ankylosing spondylitis, suggesting that there might be differences in valve performance depending on the uveitis cause [101]. Another study indicated that aqueous suppression early after surgery, when IOP is 10–15 mmHg, was associated with lower IOP later [106].

Baerveldt Valve and UG: The Baervedt Implant^®^ (BGI-250/350, Johnson & Johnson Vision, Irvine, CA, USA) has been used for more than three decades in glaucoma practice worldwide. It consists of a non-valved silicone tube attached to a silicon plate of 250 mm^2^ or 350 mm^2^ total surface. The implant is placed under two recti muscles (usually superior and lateral) and the absence of any internal valve mechanism requires additional surgical steps to restrict aqueous flow during the early postoperative period. BGI was extensively used and evaluated in a tube vs. trabeculectomy study (TVT) and primary TVT study where surgery was performed in either glaucoma patients with previous glaucoma and/or cataract surgery (TVT) or in patients with no prior incisional surgery (PTVT). BGI surgery and trabeculectomy with MMC produced similar IOPs at 5 years postoperatively in both studies while tube shunt surgery had a higher success rate (TVT), suggesting BGI’s good performance in a wide range of glaucoma patients [107,108]. Tan et al. reported results after using BGI in 47 eyes with UG. With an upper limit of 18 mmHg, the qualified success was 87% and 74% at 1 and 5 years, respectively [109]. The presence of a tube did not prevent IOP spikes during inflammation. Tan et al. reported a high rate of corneal decompensation (9%) and hypotony maculopathy (11%) as well. Chambra et al. reported a 76% qualified success rate at 5 years [110]. Casana compared the results of BGI implantation in 24 eyes with UG and 38 eyes with other forms of glaucoma. The median follow-up period was 592 days for UG and 764 days for other forms of glaucoma. At the end of follow-up time, 52.5% of UG and 32.5% of other glaucoma cases showed qualified success [111]. Manako et al. have recently reported significant IOP reductions from around 30 mmHg to 15 mmHg 1 year postoperation following BGI surgery in UG patients. This corresponded to a 1-year success rate of 88% [112]. The authors reported that the use of immunosuppressive treatment (that indicated a strong inflammatory response) was a risk factor for failure.

It is difficult to compare efficacy between different types of aqueous shunts in UG. Data from the Ahmed–Baerveldt comparison study group showed a higher vision-threatening complication rate in the BGI group at a 5-year follow up, but authors included all types of refractory glaucomas [113]. Several studies compared AGV and BGI in the management of UG. Shisha et al. compared results after performing AGV and BGI implantation in 122 eyes and concluded that after a mean follow-up of 29.6 ± 3.6 months in the AGV group and 33.1 ± 3.8 months in the BGI group, the BGI group had a greater IOP reduction (60.3% vs. 44.5%) and complete success rate (30% vs. 9%) with a higher complication rate (51.4% vs. 20.9%). The glaucoma reoperation rate was significantly higher in the AGV group (19% in the AGV group and 4% in the BGI group). Hypotony resulted in failure in 7 eyes (10%) in the BGI group and none in the AGV group [114]. The same group reported a greater incidence of corneal complications in BGI compared to AGV. Previous trabeculectomy was considered a risk factor for corneal decompensation [115].

Molteno Valve and UG: The Molteno^®^ Implant (IOP, Inc., Costa Mesa, CA, USA/Molteno Ophthalmic Limited, Dunedin, New Zealand) is a non-valved device which consists of a silicon tube attached to a single or a double plate. The double-plate model provides more surface for aqueous humour drainage but implantation is considered surgically demanding. Molteno implants have also been used for UG patients even if this is not the most frequently inserted valve. Vuori et al. reported an 85% success rate after 4 years [116].

Recently, Garagani et al. reported the results of tube implantation (mostly Molteno) in 50 eyes of 36 children with UG. Success rates were 98% at 1 year, 87% at 5 years, and 59% at 15 years; postoperative complications occurred in 36% of patients and included hypotony (22%), tube exposure (6%), tube obstruction (4%), corneal decompensation (2%) and cystoid macular edema (2%) [117].

The question of whether UG patients lose visual acuity and/or the visual field deteriorates postoperatively needs to be addressed. Vision loss in UG patients is multifactorial and can be associated not only with IOP control but also with the level of inflammation, cataract/macula status and ciliary body function. Tan et al. reported that approximately 1/3 of BGI patients suffered significant vision loss (mean follow-up: 63.6 months) [109]. Earlier reports estimated the rate to be no more than 26% after AGV implantation but the follow-up period was shorter [97,99]. A tendency of visual field loss over the first 2 years postoperation with further stabilization has been observed in BGI patients, but contemporary literature provides insufficient data regarding visual field deterioration. The long term (>2 years) outcomes of tubes according to the recent literature are presented in Table 2.

## 9. Comparison of Tubes to Trabeculectomy

The choice of a surgical procedure for UG is not an easy task. The benefits of tube surgery over trabeculectomy remains a matter of debate. Various studies have compared the two procedures. The most reported complications have been hypotony, corneal edema and hyphema for tube implantation and aqueous leakage, macular edema and cataract progression for trabeculectomy [121]. Initially, Bettis et al., in a retrospective study of 41 eyes, reported that AGV had a higher success rate compared to trabeculectomy (100% vs. 66.7% after 1 year). Most trabeculectomies failed because of relapse of the inflammation [100]. Similar results were reported by Iverson et al., who compared BGI to trabeculectomy [122]. Later studies failed to detect a difference in the success rate [69,104]. Chow et al. [104] reported significantly worse IOP control and a higher number of antiglaucoma medications in the AGV group, compared with the trabeculectomy and BGI group. Lee et al. found that trabeculectomy had a significant benefit over AGV implantation; namely, its lower postoperative IOP values, as achieved with significantly fewer antiglaucoma medications [121].

Recently, El-Saied HMA et al. prospectively compared three surgical modalities for treatment of UG in a total of 105 patients: trabeculectomy AGV implantation and trans-scleral diode laser cyclophotocoagulation. They concluded that the three modalities had the same efficacy in reducing IOP and no significant difference in complications. After 2 years, complete success was achieved in 60% via trabeculectomy, 68.6% via AGV and 62.9% via TD-CPC [123].

Nevertheless, according to most experts, certain situations most clearly call for a tube as the first surgical intervention. These include patients with active inflammation at the time of surgery as well as those with other known risk factors for trabeculectomy failure: young age, black race, aphakia or pseudophakia and prior failed glaucoma surgery.

No matter which surgical approach is elected, up to 1/3 of UG will need a second or even a third operation. The activity of postoperative inflammation may be a critical factor for the longevity of the procedure [124].

## 10. Management Algorithm in a Patient with Uveitic Glaucoma

Initial treatment for UG should target the cause of uveitis (especially in cases of infectious uveitis), the inflammation and the IOP. PCR to detect viral DNA may be performed in cases of anterior uveitis with a PSS or Fuchs phenotype to detect CMV, as specific antiviral treatments may significantly improve the prognosis of glaucoma. In the follow-up of UG glaucoma patients’ visual fields, disc photos should not be omitted along with OCT. The value of OCT angiography should be verified with further studies. Gonioscopy should define whether there is angle closure and indicate the need of laser peripheral iridotomy in cases of angle-closure glaucoma. Any class of antihypertensive medication (except cholinergic agonists) may be used for IOP control. SLT may only be considered in cases of quiescent inflammation when steroid response is suspected. Once surgical intervention for medically uncontrolled IOP is deemed necessary, glaucoma and uveitis specialists must coordinate to succeed in this task. Ocular surgery will produce a significant amount of inflammation, regardless of the adequate control that may have been achieved preoperatively. Additional perioperative suppression of the inflammatory cascade that the operation will generate is mandatory. There are no large-scale studies providing a specific algorithm; however, in our practice, the type of uveitis (anterior or posterior) and the means to achieve quiescence employed in the past (from the patient’s medical records) is reviewed, in order to decide upon the best perioperative approach. A common agreement suggests that either topical, periocular or systemic steroids should be introduced a few days prior to surgery, according to the severity of previous ocular inflammation, and a slow taper should follow postoperatively. A difficult subject is the need for glaucoma surgery in eyes for which uveitis control is suboptimal; however, in real-world situations, the quiescent period may be small or even absent, or a low-grade inflammation may persist despite maximum treatment. These situations are not well described in literature and a leap of faith may be required. There is no unanimity regarding the best surgical approach, and trabeculectomy, tubes or even MIGS may be tried depending on the clinical picture and the preference of the surgeon [85].

## 11. Future Directions

In recent years, many advances have occurred in the diagnosis and especially the treatment of UG. However, there are various subjects that need to be clarified by further studies. The value of OCT angiography in the follow up of UG has not been adequately studied. The place of MIGS in the surgical algorithm of UG should also be assessed. The best surgical treatment of UG according to the clinical scenario (anterior vs. posterior uveitis, active vs. inactive, open angle glaucoma vs. angle-closure glaucoma) should also be defined. Finally, given that a recent prospective study regarding UG treatment indicated that TD-CPC is not inferior to surgical treatment, we should reassess its role in UG treatment, especially in the micropulse mode.

## 12. Conclusions

Uveitic glaucoma is a complex disease. Many pathogenic mechanisms are involved alone or in combination. The diagnostic approach is problematic as inflammatory activity may affect the interpretation of diagnostic and staging tests. In turn, the treatment poses a lot of dilemmas. Intraocular inflammation along with IOP should be controlled and the benefits of steroid treatment should be carefully balanced with the risks. The young age of glaucoma patients warrants a stepwise approach. Conservative and conjunctival sparing surgical approaches should be adopted. Minimally invasive surgical approaches have been proven effective and are increasingly being adopted in the management of UG. Whether indicated, either trabeculectomy or a tube may be equally effective depending on the condition of the patient and the preference of the doctor.

## Figures and Tables

**Table 1:** Recent studies reporting long term (>2 years) outcomes of surgical treatment in adult uveitic glaucoma.

	Procedure	Patients/ Eyes	Diagnosis (%)	F/U (Months)	Success Rate (%) *	Complications	Secondary Procedures (%)
Almobarac (2017) [64]	Trabeculectomy	50/70	VKH 38.6% Idiopathic 24.3 Fuchs 15.7	77 ± 40.9	35.7	Cataract 45.3 Hypotony 30 IOP spike 10	25.7
Kanaya (2021) [65]	Trabeculectomy	50/50	Idiopathic 42 Sarcoidosis 34 VKH 8 Bechset 8	120	66.5	Hyphema 14 Choroidals 14	16
Known (2017) [69]	Trabeculectomy	54/54	Idiopathic 42 Fuchs 14 Herpetic 8.5 Sarcoidosis 8.5	24 ± 21	67	Late hypotony 15	
	BGI	28/28		31 ± 21	75	Late hypotony 11 Corneal edema 3.6 Endophthalmitis 7.1	
Chen (2023) [78]	KDB	22/24	Idiopathic anterior 45 idiopathic posterior 17.5	24	69	Cyclodialysis cleft 8.3 Transients IOP elevation 17 Early hypotony 8	12.5
	GATT	40/33		24	80	Cyclodialysis cleft 3 Transient IOP elevation 20 Hyphema 10	7.5
Triolo (2023) [92]	Preserflo	21/21	Idiopathic 52 Possner Schlosman 10 Sarcoidosis 10		47	Button hole 4.7	57.1
Merceica (2017) [94]	Deep sclerctomy	43/43	Idiopathic 35 Fuchs 32.5 Herpes 9.3	68 ± 33	60	Transient hypotonoous maculopathy 5 Vision loss 2	16.3
Salloukh (2021) [96]	Viscocanalostomy	16		60	75	Transient IOP > 30.73	25

(*): according to the definition by the author; VKH: Vogt–Koyanagi–Harada; BGI: Baerveldt glaucoma implant; KDB: Kahook dual blade; GATT: gonioscopy-assisted transluminal trabeculotomy; JIA: juvenile idiopathic arthritis.

**Table 2:** Recent studies reporting long term (>2 years) outcomes of tubes in adult uveitic glaucoma.

	Procedure	Patients/ Eyes	Diagnosis (%)	F/U (Months)	Success Rate * (%)	Complications (%)	Secondary Procedures (%)
Kubaisi (2017) [118]	AGV + FAI	9/10	Panuveitis 44.4			RD 10	
Bao (2018) [102]	AGV	57/66	Unknown 35.8 Ankylosing spondylitis 31.3	53.3 ± 8.5	61.2	Tube erosion 7.6% RD 3%	40
Voykov (2018) [119]	AGV	17	Fuchs	36	38.4	Tube erosion 23.5 Tube obstruction 23.5 Diplopia 4	35
Yakin (2017) [120]	AGV	35/47	ABD	57.7 ± 26.1	35.9	Tube erosion 3.2 Early wound dehiscence 2.1	6.4
Sungur (2017) [101]	AGV	39/46	Idiopathic anterior uveitis 17.3 Ankylosing spondilitis 17.3 Fuchs 17.3	51.9 ± 23	63	Tube exposure 2.2	
Tan (2018) [109]	BGI	47/47	Unknown 30 Fuchs 17 Sarcoidosis 13	63.6 ± 43.1	75	Cornea decompensation 9 Hypotony maculopathy 11	17
Chambra (2019) [110]	BGI	42/34	Sarcoidosis 31 Idiopathic 21 Tuberculosis 14	58.5. ± 20.8	76	CME 5 Hypotony maculopathy 2	31
Sisha (2020) [114]	AGV	67	Anterior uveitis 86 Panuveitis 7 Posterior 6	30 ± 4	63	Tube exposure 12	45
Sisha (2020) [114]	BGI	70		33 ± 4	70	Corneal edema 6 Tube exposure 4 Endophthalmitis 1	59

(*): according to the definition by the author; AGV: Ahmed Glaucoma Valve; FAI: fluocinolone implant; BGI: Baerveldt glaucoma implant; ABD: Adamantiades–Behcet’s disease; RD: retinal detachment.

## Data Availability

Not applicable.
